# Beyond the Binary: On the Multiplicity of Sex and Gender in a Western-Centric World

**DOI:** 10.1007/s10508-025-03157-8

**Published:** 2025-08-12

**Authors:** Joel R. Anderson

**Affiliations:** Australian Research Centre in Sex, Health and Society, La Trobe University, NR6, Melbourne, 3806 Australia

How many sexes are there? How many genders? These deceptively simple questions are often answered with rigid binaries: two sexes (male and female) and two genders (man and woman). Yet, such answers reflect a specifically Western, colonial framework rather than biological or cultural reality. Trans-inclusive and cross-cultural perspectives consistently show that binary sex and gender models are insufficient to capture the full spectrum of human diversity (Fausto-Sterling, 2008; Hyde et al., 2019; Morgenroth & Ryan, 2018). In addition, sex/gender dichotomous thinking is known to drive prejudices toward gender diverse people as well as sexual prejudice and femmephobia (Campbell et al., 2018; Falomir-Pichastor et al., 2019; Hoskin, 2020; Prusaczyk & Hodson, 2020). In this commentary, I critically examine these questions, arguing that Western-centric binary thinking conflates sex and gender, erases cultural variation, and undermines the lives and identities of trans and nonbinary people.

## Sex as a Spectrum, Not a Binary

In Western science, sex has consistently been portrayed as a binary and biologically determined construct, but contemporary research reveals that sex is multifaceted and best understood as a spectrum. For instance, people with innate variations to their sex characteristics (sometimes called “intersex”) have chromosomes, hormones, or genitalia that do not fit binary definitions. Their prevalence is estimated to be around 1.7% of the population (Ainsworth, 2015), which is roughly as common as having red hair. One important consideration about the rigidity of conceptualizing sexes is how an infant is assigned their sex. Sex assignment at birth typically relies on visible genitalia, which is often a reliable indicator. However, other dimensions of sex (e.g., chromosomes, internal reproductive structures) may contradict this surface classification. For example, people with complete androgen insensitivity syndrome have XY chromosomes (which is the chromosomal structure used to presume an infant male at birth), but they may develop female phenotypes and often identify as women (Fausto-Sterling, 2008). These cases of biological variations disrupt the coherence of arguments about binary sex and expose its role as a social classification system, not merely a natural fact. Together, these well-documented examples challenge the idea that male and female are the only "natural" sexes.

In addition, some scholars argue that sex is binary based on the type of gametes a body is organized to produce—sperm or ova—and that intersex variations are atypical developments within an underlying male or female structure (e.g., Byrne, 2023; Wright, 2023). I argue that this position emphasizes reproductive roles and overlooks the lived realities and complex embodiment of those whose sex characteristics do not align neatly with binary classifications. It also fails to account for the fact that many people—intersex or not—do not and cannot produce gametes (e.g., due to age, surgery, or medical conditions), yet we do not question their sex. Defining sex solely by gamete production narrows it to a hypothetical reproductive function, rather than acknowledging the diverse ways sex is biologically and socially experienced. As such, gamete-based definitions are overly reductive and ill-suited to capture the full complexity of human variation.

Western societies have historically enforced the sex binary through medical interventions. Intersex infants have been subjected to nonconsensual surgeries to "normalize" their bodies, reinforcing binary norms at the cost of bodily autonomy (Hegarty, 2023). These practices reflect an ideological investment in the binary, not a biological imperative. The implications of binary sex classifications reach beyond the clinical. Legal and institutional structures across the West rely on binary categories, resulting in systemic erasure of intersex individuals. From gender markers on identification documents to rigid sex-based categorizations in education and sport, institutional frameworks uphold a binary logic that harms those who do not fit its mold. This logic often positions nonbinary sex as an aberration to be managed, erased, or corrected.

## Gender Beyond the Binary

Gender, like sex, is also typically framed in binary terms. Western cultures conflate sex with gender, assuming that two sexes necessitate two corresponding gender identities—and this conflation occurs in research, social and family environments, medical and government records, and is perpetuated in government policies (Bittner & Goodyear-Grant, 2017; Lau et al., 2020; Lyons et al., 2020, 2021). However, anthropological research documents gender diversity across cultures. The hijra of South Asia, fa'afafine of Samoa, two-spirit identities in many Indigenous North American communities, and the five genders of the Bugis in Indonesia all illustrate that nonbinary gender categories are historically and culturally recognized (Hussain, 2024; Nanda, 2014; Satrianegara et al., 2021; Sylliboy, 2021; Vasey & Bartlett, 2007). These systems were often suppressed through colonial imposition of Western norms. For instance, British colonial rule criminalized the Hijra community in 1871, labeling them as "eunuchs" and stripping them of civil rights (Hinchy, 2017). Similarly, two-spirit traditions in Canada and Northern America were violently erased through Christian mission schools and anti-sodomy laws (Driskill, 2010). These erasures were not accidental—they were integral to colonial domination: By imposing a binary gender system, colonizers dismantled Indigenous social structures and spiritual traditions. Lugones (2007) characterized this as the colonial/modern gender system—a racialized and hierarchical system that imposed binary gender and heterosexualism as universal truths. The coloniality of gender continues to shape contemporary Western institutions, reinforcing gender binaries in ways that often obscure their historical and cultural contingency.

## The Conflation of Sex and Gender in Western Thought

Western epistemologies have historically conflated sex and gender, pathologizing any departure from assigned categories. The belief in a stable, binary mapping of gender onto sex is both scientifically flawed and politically harmful. Butler (1990) famously argued that gender is performative, not innate, and that it is enacted through repeated behaviors rather than emerging naturally from biology. This performative view undermines the assumed naturalness of gender categories and invites a more fluid understanding of identity. Yet, this nuance has been resisted in both policy and public discourse. During the first Trump administration, for instance, efforts were made to define gender strictly "as determined by biological sex at birth" (Green et al., 2018) and during his more recent second administration, an executive order was issued stating that the government of the USA will recognize only two sexes (male and female) and that these are fixed at birth (Thoreson, 2025). These actions each effectively erased trans identities and reasserted a rigid binary. This redefinition has far-reaching implications, threatening the legal recognition and healthcare access of trans and nonbinary people.

Such policies are symptomatic of a broader pattern in Western political discourse: a reliance on biological essentialism to police and restrict gender diversity. By invoking "science" to support a binary model, these policies misrepresent scientific consensus, which increasingly supports nonbinary understandings of sex and gender (Hyde et al., 2019).

## Case Studies in Gender Diversity and Resistance

Despite systemic attempts to enforce the binary, communities around the world have maintained or reclaimed nonbinary gender roles. For example, two-spirit resurgence in Indigenous North America is a powerful act of cultural recovery and resistance. Activists and scholars have revitalized two-spirit identities not only as a matter of gender diversity, but also as a reclamation of Indigenous knowledge systems disrupted by colonization (Driskill, 2010). In South Asia, hijra communities continue to resist marginalization through kinship structures, cultural rituals, and legal advocacy. India legally recognized a third gender in 2014, a move influenced by hijra activism and legal challenges that invoked constitutional rights to dignity and equality (Hinchy, 2017). In Indonesia, Bugis gender categories persist in some rural communities despite state Islamization and pressures toward binary conformity (Satrianegara et al., 2021).

These case studies demonstrate that gender diversity is not a recent invention or a Western anomaly. On the contrary, gender multiplicity has been a part of human societies for centuries. What is recent is the global imposition of binary models under the guise of modernity and progress.

## Toward a Trans-Inclusive, Decolonial Understanding

A trans-inclusive approach requires decoupling sex from gender and recognizing both as complex, socially situated phenomena. It also demands decolonizing our frameworks: acknowledging that the binary is not universal, but a product of Western colonialism and white supremacy (Lugones, 2007; Oyewumi, 1997). Decolonizing gender involves questioning the authority of Western knowledge systems and elevating alternative epistemologies. For example, Oyewumi (1997) argued that Yoruba society in precolonial Nigeria did not organize social life around gender, but rather around seniority and kinship. The imposition of Western gender categories through colonial rule thus introduced an alien framework that reshaped Yoruba social relations and subordinated women. Similarly, Lugones (2007) critiqued the export of the European gender binary as a tool of colonial violence. Under this system, colonized women were denied the status of "women" altogether, while colonized men were emasculated through systemic exclusion from patriarchal privilege. Recognizing these histories is crucial to understanding how deeply the gender binary is embedded in colonial hierarchies.

Today, more than a dozen countries recognize a third gender option and psychological science increasingly supports the abandonment of binary models in favor of dimensional approaches (Hyde et al., 2019). These changes signal a global shift, though resistance remains strong. Trans and nonbinary people continue to face institutional erasure and violence, particularly in societies where the binary is culturally dominant and politically weaponized. Efforts to resist these forces are visible in grassroots activism, legal reform, and academic scholarship. Trans activists and scholars have expanded public understandings of gender (as a unique construct from sex) through storytelling, education, and policy advocacy. Online communities have also played a key role in connecting individuals across borders and building movements that challenge gender essentialism.

## Conclusion

The question is not simply how many sexes or genders exist, but why Western societies have so aggressively limited the answers to two. A more accurate, just, and inclusive view acknowledges that sex is a biological spectrum and gender is a cultural, psychological, and political construct that varies across time and space. Rather than count genders, we should affirm the right of individuals to define and express their own. Only by rejecting binary frameworks and embracing plurality can we begin to undo the harms of gender essentialism and build a world where all identities are legible and livable.
